# Human intracranial recordings reveal distinct cortical activity patterns during invasive and non-invasive vagus nerve stimulation

**DOI:** 10.1038/s41598-021-02307-x

**Published:** 2021-11-23

**Authors:** William L. Schuerman, Kirill V. Nourski, Ariane E. Rhone, Matthew A. Howard, Edward F. Chang, Matthew K. Leonard

**Affiliations:** 1 Department of Neurological Surgery, University of California, San Francisco, 505 Parnassus Ave, Room M779, Box 0112, San Francisco, CA 94143 USA; 2 Weill Institute for Neurosciences, University of California, San Francisco, 505 Parnassus Ave, Room M779, Box 0112, San Francisco, CA 94143 USA; 3 Department of Neurosurgery, The University of Iowa, Roy J. and Lucille A. Carver College of Medicine, 200 Hawkins Drive, Iowa City, IA 52242-1089 USA; 4 Iowa Neuroscience Institute, 169 Newton Road, 2312 Pappajohn Biomedical Discovery Building, Iowa City, IA 52242 USA; 5 Pappajohn Biomedical Institute, 169 Newton Road, 6332 Pappajohn Biomedical Discovery Building, Iowa City, IA 52242 USA

**Keywords:** Neuroscience, Neurology, Neurophysiology

## Abstract

Vagus nerve stimulation (VNS) is being used increasingly to treat a wide array of diseases and disorders. This growth is driven in part by the putative ability to stimulate the nerve non-invasively. Despite decades of use and a rapidly expanding application space, we lack a complete understanding of the acute effects of VNS on human cortical neurophysiology. Here, we investigated cortical responses to sub-perceptual threshold cervical implanted (iVNS) and transcutaneous auricular (taVNS) vagus nerve stimulation using intracranial neurophysiological recordings in human epilepsy patients. To understand the areas that are modulated by VNS and how they differ depending on invasiveness and stimulation parameters, we compared VNS-evoked neural activity across a range of stimulation modalities, frequencies, and amplitudes. Using comparable stimulation parameters, both iVNS and taVNS caused subtle changes in low-frequency power across broad cortical networks, which were not the same across modalities and were highly variable across participants. However, within at least some individuals, it may be possible to elicit similar responses across modalities using distinct sets of stimulation parameters. These results demonstrate that both invasive and non-invasive VNS cause evoked changes in activity across a set of highly distributed cortical networks that are relevant to a diverse array of clinical, rehabilitative, and enhancement applications.

## Introduction

Vagus nerve stimulation (VNS) is being used increasingly as a treatment for conditions^1^ such as epilepsy^2^, depression^3^, and chronic inflammation^4^, as an adjuvant to rehabilitation^5^, and also as a technique for cognitive enhancement^6^. The recent rapid expansion of the VNS application space has been driven in part by the putative ability to stimulate the nerve non-invasively, creating the opportunity for novel uses in healthy populations^7^. While VNS typically refers to stimulation using a cuff electrode implanted at the main cervical branch of the vagus nerve located in the neck (iVNS), transcutaneous vagus nerve stimulation can be performed at the skin on the neck (targeting the cervical branch) or the ear^8^ (targeting the auricular branch of the vagus nerve: taVNS). Though differences exist between the morphology and anatomical pathways of the main cervical branch, which is a mixed cranial nerve composed of efferent (20%) and afferent (80%) nerve fibers^9^, and the auricular branch, which is purely afferent and exhibits complex connectivity^10,11^, taVNS may have the potential to achieve similar effects as iVNS without the need for surgery^12^.

Although VNS has been shown to lead to positive outcomes in many cases, efficacy is highly variable and difficult to predict^13–15^. Despite decades of use and numerous studies into its mechanisms, we still lack a clear understanding of how VNS affects basic human neurophysiological activity in target brain structures and how such changes in activity may vary with respect to commonly used stimulation parameters^16^. In addition to potentially limiting the efficacy of VNS, this knowledge gap complicates comparisons between implanted and non-surgical stimulation techniques.

While there have been prior investigations into the cortical and subcortical effects of iVNS and taVNS using methods like electroencephalography (EEG) and functional magnetic resonance imaging (fMRI), these studies have produced conflicting results. For example, while early EEG studies employing a range of stimulation frequencies reported no effects^17,18^, more recent work suggests that 10 Hz VNS can produce acute changes in low frequency EEG power^4^. Non-invasive neuroimaging of cerebral blood oxygenation during iVNS and taVNS has also produced mixed results^19^, with differences in both the direction and location of the effects. There have also been a small number of intracranial electroencephalography (iEEG) studies that have benefited from the superior signal-to-noise ratio of direct cortical recordings, but these have also produced inconsistent results. In two recent iEEG studies on acute iVNS that used similar stimulation parameters, one reported decreases in low-frequency spectral band power and no changes in high frequency broadband power^20^, while the other reported the opposite^21^. Similarly, an iEEG study investigating the effects of VNS amplitude on functional connectivity reported distinct patterns of effects across individuals^22^. Two possible reasons for the high variability among these studies are (1) a lack of high spatial and temporal resolution to characterize relatively subtle effects—even the iEEG studies mentioned above are limited to a small number of recording channels—and (2) a need to investigate the effects of acute VNS on human physiology at the individual participant level.

**Figure 1:**
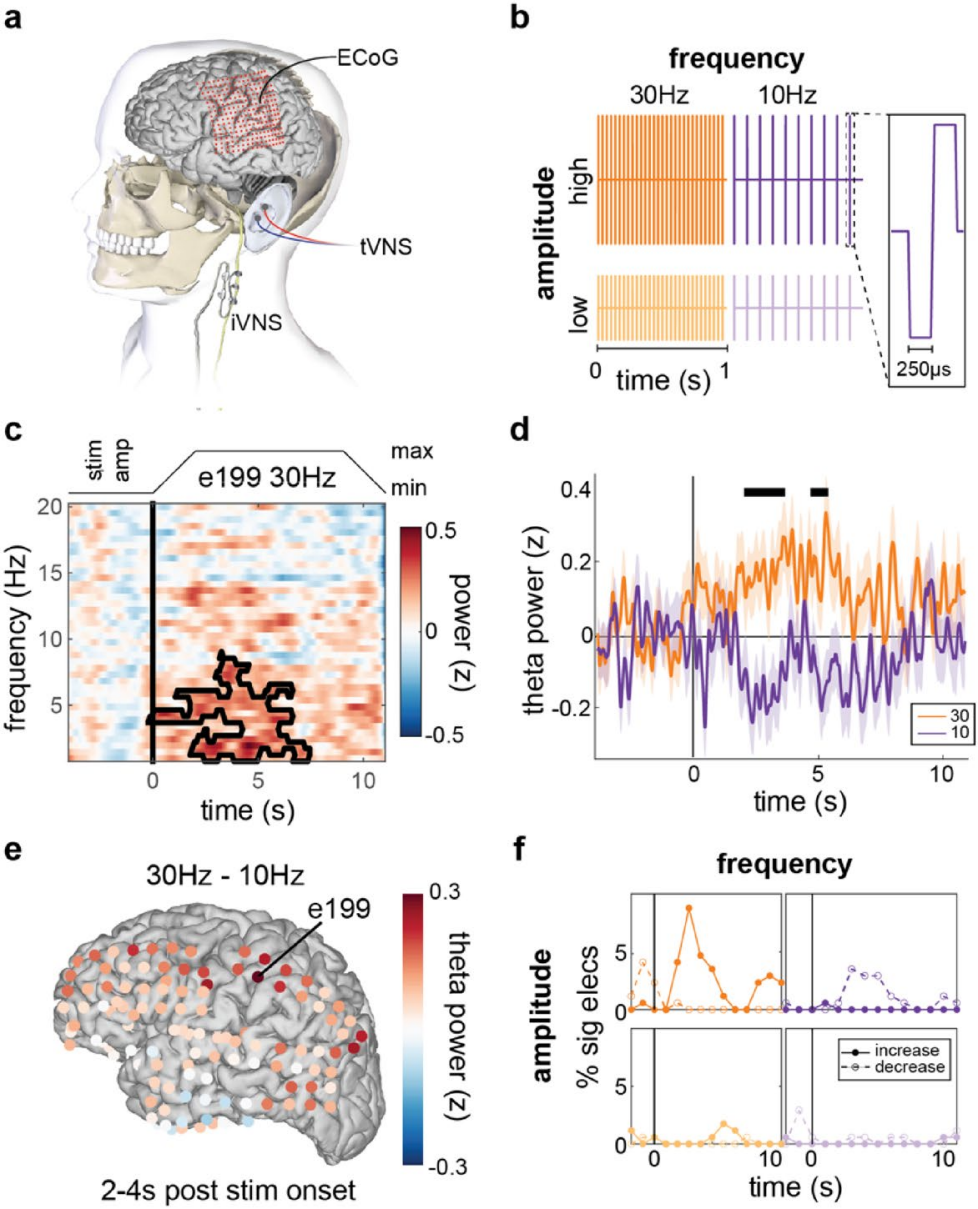
Implanted VNS (iVNS) evokes small, widespread changes in low frequency spectral band amplitude dependent on stimulation parameters. (**a**) The vagus nerve can be stimulated directly via an implanted pulse generator and a cuff electrode around the left cervical vagus (iVNS). Electrodes can also be placed on skin of the outer ear for transcutaneous auricular VNS (taVNS). (**b**) VNS was delivered at two levels of frequency (30 Hz and 10 Hz) and two levels of amplitude (below each participant’s perceptual threshold [high] and half of that level [low]). Stimulation consisted of trains of biphasic square wave pulses (inset). (**c**) Average neural spectrogram for a single electrode time-locked to onset of 30 Hz iVNS in one example participant (P1). Black boundary line indicates a spectro-temporal cluster with increased amplitude relative to baseline. Stimulation duty cycle shown at top. (**d**) Mean and standard error of Z-scored analytic amplitude in the Theta band (4–8 Hz) for the same electrode as in (**c**). Black lines denote temporal clusters corresponding to significant differences between 30 and 10 Hz trials. (**e**) Difference in average theta amplitude between 30 Hz or 10 Hz iVNS for each electrode from 2 to 4 s after stimulation onset. Greater differences between conditions found across anterior frontal, parietal, and posterior temporal/occipital electrodes. (**f**) For each stimulation setting (**b**), the percentage of electrodes showing significant (p < 0.01, uncorrected) theta amplitude increases (solid) or decreases (dashed) relative to baseline within each 2-s window (1 s overlap) time-locked to stimulation onset for this example participant. Across electrodes, 10 Hz and 30 Hz stimulation appear to elicit opposed changes in amplitude, peaking around onset of maximum stimulation amplitude.

Here, we take advantage of a rare opportunity to explore the effects of VNS modalities and stimulation parameters on intracranial electrophysiology using high-density electrocorticography (ECoG) and stereotactic electroencephalography (SEEG) with depth electrodes (Fig. 1a; Supplementary Fig. S4). Patients (n = 7, Table 2) undergoing intracranial monitoring for seizure activity, a subset of whom had iVNS devices (n = 3), volunteered to undergo short sessions of VNS while they sat quietly. We employed three different stimulation modalities: iVNS delivered with a rapid duty cycle^23^, taVNS delivered with the same rapid duty cycle (taVNS-matched), and ‘short burst’ taVNS stimulation consisting of 15 pulses delivered with no ramp (taVNS-short). taVNS-short has been used in many neuromodulation studies and is of great interest for applications requiring fine temporal precision between stimulation and behavior^24–27^. For each of these modalities, we varied the stimulation frequency and amplitude, and examined neurophysiological responses time-locked to the onset of VNS delivered below individual participant perceptual thresholds (which we refer to as evoked activity). We focused our analyses on characterizing acute VNS-evoked changes in low-frequency spectral band power within individuals, with both qualitative and quantitative comparisons across the participant sample to examine inter-individual variability.

This design allowed us to answer three questions that are fundamental to our understanding of VNS: (1) How are local neural populations and cortical networks acutely modulated by iVNS and taVNS? (2) How do different stimulation parameters modulate cortical activity and how do such patterns vary across modalities and individuals? (3) How similar are iVNS and taVNS, particularly when comparing matched stimulation parameters and commonly-used short bursts of pulses? By investigating these questions, we aim to provide a timely, empirical description of the effects of specific VNS parameters and modalities on cortical neurophysiology at the level of the group and the individual.

## Results

We first conducted exploratory analyses targeting low-frequency spectral power time-locked to the onset of iVNS (which we refer to as ‘iVNS-evoked’ responses) in an example participant (P1). In addition to having extensive ECoG coverage spanning multiple cortical regions, this participant received stimulation in all three modalities (iVNS, taVNS-matched, and taVNS-short), enabling direct comparison between traditional iVNS (direct stimulation of the ascending cervical vagus) and the more recently developed taVNS modalities (Fig. 1a). Focusing first on single electrodes, we identify potential spectrotemporal patterns of activity evoked by iVNS delivered with different stimulation parameters. We then characterize the robustness of these effects across all electrodes. In later analyses, we characterize the variability of these effects across participants.

### In response to iVNS, local neural populations exhibit small, parametrically-modulated changes in low frequency power across widespread cortical and subcortical regions

Figure 1 depicts the results of the exploratory single electrode analyses. Stimulation in the iVNS modality consisted of 11-s pulse trains (square biphasic pulses; 2-s onset/offset ramp periods) followed by an 8 s rest period. In each experimental block we delivered stimulation using a specific combination of stimulation parameters (frequency and amplitude; Fig. 1b). The duration of each block was approximately 10 min (~ 30 trials).

For all ECoG and depth electrodes, we extracted the amplitude of neural activity from 1 to 20 Hz. For each trial and spectral band, power was normalized to the mean of the pre-trial baseline period (− 4 to − 0.5 s) and the standard deviation of the entire trial^28^. Visual inspection of the spectrotemporal responses time-locked to onset of stimulation revealed electrodes in which specific iVNS parameters appeared to elicit changes in power relative to baseline (e.g., e199, Fig. 1c). A two-dimensional cluster-based permutation test (CBPT)^29^ on 30 Hz trials in this electrode revealed a single significant cluster (p < 0.0001). While not denoting the exact boundaries of the effect^30^, the cluster had a spectral range of 1–8.8 Hz (corresponding roughly to canonical delta and theta ranges) and a temporal range beginning at the onset of stimulation and lasting for approximately seven seconds. The magnitude of this evoked change was relatively small (mean z-score in cluster = 0.28) but on the same scale as effects observed with direct cortical stimulation during restful waking that have been associated with changes in mood^31^. The same test on 10 Hz trials revealed one trend (p = 0.051) associated with a *decrease* in power (mean z-score = − 0.25), suggesting that stimulation parameters may elicit opposing changes in low-frequency power.

To test whether different stimulation parameters caused different evoked changes in low-frequency neural activity, we directly compared stimulation levels in three canonical spectral bands: delta (1–4 Hz), theta (4–8 Hz), and alpha (8–12 Hz). First, we evaluated the effect of stimulation frequency in the same example electrode for the theta band. Between approximately 2–6 s following stimulation onset, average theta power increased for 30 Hz iVNS and decreased for 10 Hz iVNS (Fig. 1d) and a cluster-based permutation test between 30 and 10 Hz trials found two temporal clusters (2.09–3.76 s, p < 0.0001; 4.8–5.52 s, p = 0.013), the first of which corresponded roughly to the time when the stimulation amplitude reached its peak (see Fig. 1c, top). No significant effect of amplitude was found in this electrode.

The single electrode analysis showed that 30 Hz iVNS elicited increases in theta power while 10 Hz iVNS elicited decreases, and this occurred approximately when stimulation reached peak amplitude (t = 2 s). To examine the topography of these differences, we averaged power in a two-second window (2 s to 4 s) for all 30 Hz trials and all 10 Hz trials. Plotting the difference between the two, a broad spatial pattern emerged comprising frontal, parietal, and temporal-occipital electrodes (Fig. 1e). To quantify the consistency of increases/decreases in power across all electrodes and all stimulation conditions, for each combination of stimulation frequency and amplitude we averaged power within two-second windows (1 s overlap) and applied non-parametric rank sum tests. No electrodes remained significant after correcting for multiple comparisons. For illustrative purposes, we set an arbitrary alpha of 0.01 and with this cutoff observed an increase in the proportion of electrodes eliciting a frequency-specific increase/decrease in power beginning around onset of peak stimulation, but only for high amplitude trials (Fig. 1f).

Together, these results demonstrate that in this participant, iVNS caused small, temporally-specific changes in cortical theta power in local neural populations (Fig. 1c,d) and across multiple brain regions (Fig. 1e). In this stimulation modality, acute modulations of activity were driven primarily by stimulation frequency (Fig. 1d,f).

### iVNS-evoked activity occurs at the level of broad spatiotemporal networks

In the example participant, we observed parameter-specific evoked effects of iVNS in single electrodes. However, these patterns were relatively small (Fig. 1d), rare (Fig. 1f), and highly distributed (Fig. 1e), suggesting that VNS may modulate neural activity in broader networks or regions rather than specific electrodes.

**Figure 2:**
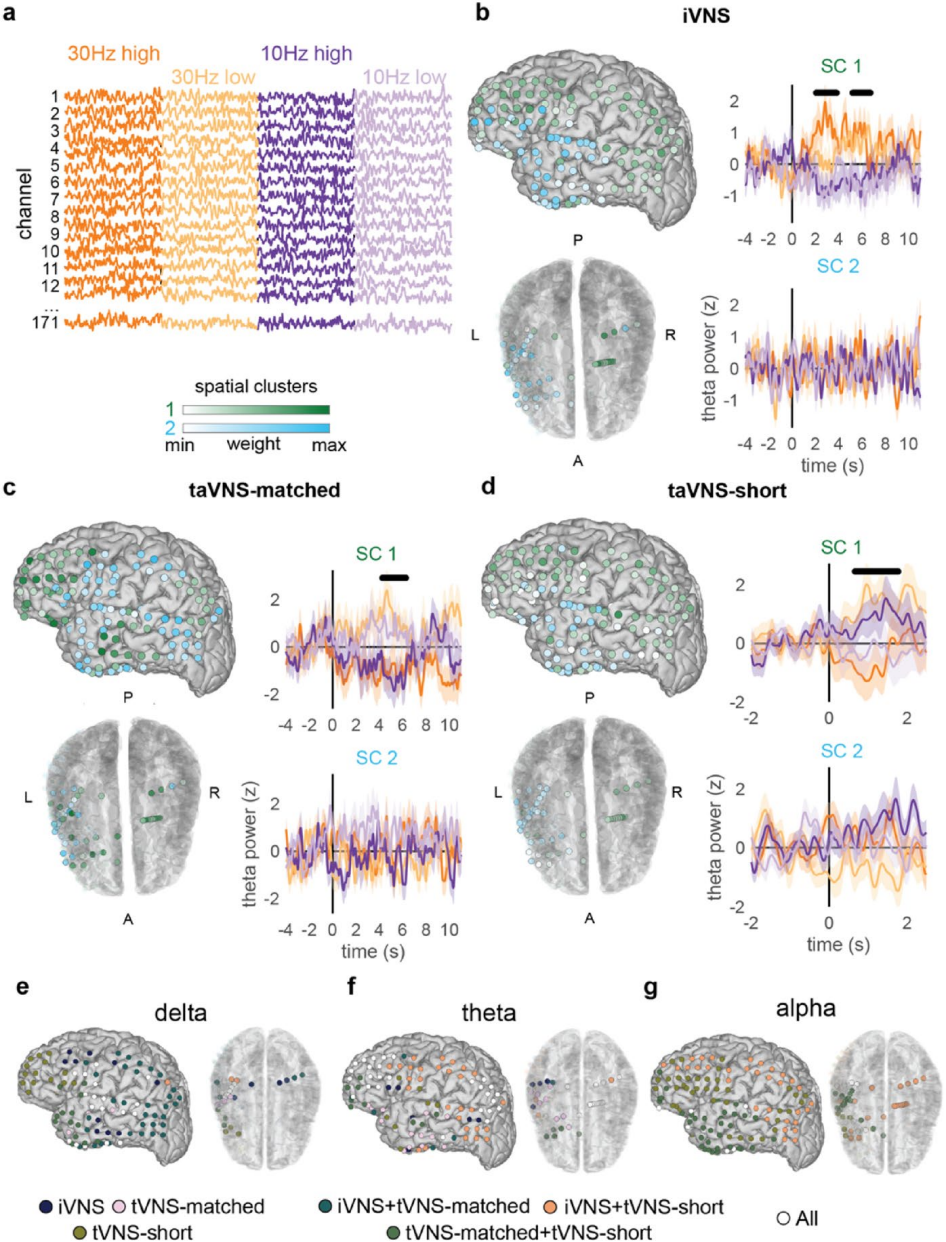
Spatiotemporal patterns of VNS-evoked theta amplitude vary between stimulation modalities. (**a**) Event-related amplitude was averaged over trials by stimulation condition and concatenated into a 2-dimensional channel × time matrix. (**b**) Unsupervised clustering of iVNS data for P1 shows two spatial clusters with correlated activity. Colors indicate cluster assignment and weighting. Timecourses of cluster-weighted theta amplitude for each spatial cluster (SC) show differences between stimulation parameters for SC1 (black bars indicate extent of temporal clusters [p < 0.05; cluster-based permutation tests]; main effect of frequency). (**c**) SCs for taVNS-matched are spatially distinct compared to iVNS. Timecourses of cluster-weighted theta amplitude show a significant effect for SC1, with a main effect of amplitude. (**d**) Clusters for taVNS-short are similar to iVNS, but again exhibit a distinct pattern of parameter-specific evoked timecourses compared to iVNS and taVNS-matched. SC1 exhibited a significant frequency x amplitude interaction effect. (**e**) Spatial overlap of significant effects between modalities for delta band power. Each plotted electrode was assigned to a cluster in which one or more significant effects was found in one or more modalities (designated by color). (**f**) Same as (**e**), for theta band power. Numerous frontal, parieto-occipital, and hippocampus/amygdala electrodes associated with significant effects in all three modalities. (**g**) Same as (**e**), for alpha band power. Extensive overlap between iVNS and tVNS-short across superior fronto-temporal and parietal electrodes, as well as temporal-occipital electrodes.

To address this question, we used unsupervised clustering (convex non-negative matrix factorization; cNMF^32^) to identify distributed patterns of neural activity that varied consistently in response to the four sets of stimulation parameters (Fig. 2a). Focusing on iVNS stimulation and theta band amplitude for illustration, we found that activity across electrodes was best described by two spatial clusters (SCs) that qualitatively matched the pattern of results observed in the single electrode analyses (Fig. 1e). SC1 was composed of electrodes widely distributed across frontal, parietal, posterior temporal, and medial temporal lobe areas. In contrast, SC2 was more confined to anterior temporal electrodes (Fig. 2b).

Projecting the cNMF weights onto the original data produced a weighted average representing activity across all electrodes for that cluster^33^. We then applied the same techniques utilized in the single electrode analyses (Fig. 1d) to test for parametric variation in activity within each spatial cluster. In SC1, we found two significant temporal clusters (TCs) that exhibited a main effect of stimulation frequency (TC1: 2.06–3.81 s, *p* = 0.015; TC2: 5.19–6.71 s, *p* = 0.017; CBPT on a 2-way ANOVA with frequency and amplitude as factors; Fig. 2b, right upper). As in the single electrode example, the frequency effect manifested as an increase in theta amplitude for 30 Hz, with either no change or a small decrease in amplitude for 10 Hz stimulation, regardless of amplitude. No significant differences between conditions were found in SC2 (Fig. 2b, right lower).

### iVNS, taVNS-matched, and taVNS-short have distinct, parameter-specific effects on cortical activity

The same procedure was applied to the taVNS-matched and taVNS-short datasets. In both cases, a rank-2 decomposition was found to be optimal. For taVNS-matched, SC1 exhibited a significant main effect of amplitude (4.26–6.27 s, *p* = 0.021; Fig. 2c, right top), with theta being greater during low amplitude stimulation compare to high amplitude stimulation. For taVNS-short, we found a frequency × amplitude interaction in SC1 (0.66–1.78 s, *p* = 0.023; Fig. 2d, right top). Specifically, increases in theta were observed for 25 Hz high amplitude stimulation and 10 Hz low amplitude stimulation, while no change/decreases in theta were observed for 25 Hz low amplitude stimulation and 10 Hz high amplitude stimulation.

Compared to iVNS, the taVNS-matched clusters were less spatially contiguous, with SC1 comprised of electrodes over anterior frontal, posterior parietal, ventral temporal, and medial temporal lobe areas, and SC2 comprised of electrodes over ventrolateral frontal, sensorimotor, posterior temporal, and anterior subtemporal cortex (Fig. 2c, left). Directly comparing cluster assignments between modalities, only 58% of electrodes were assigned to the same cluster. We quantified cluster similarity using the adjusted Rand index (ARI^34^, where an ARI of 1 indicates identical assignment and an ARI of 0 indicates similarity equal to chance) and found a very low degree of similarity between iVNS and taVNS-matched (ARI = 0.02). In contrast, the decomposition for taVNS-short was more similar to that of iVNS (73% agreement, ARI = 0.21, Fig. 2b) than taVNS-matched (63% agreement, ARI = 0.07, Fig. 2c).

To illustrate both the differences and overlap among stimulation modalities, we characterized each individual electrode according to whether it showed significant effects in iVNS, taVNS-matched, and taVNS-short. In the delta (Fig. 2e), theta (Fig. 2f), and alpha (Fig. 2g) bands, there were electrodes that showed effects only in a single modality. However, there were also a large number of electrodes that showed effects in more than one modality, including a network of frontal and parietal electrodes that showed effects in all three modalities in theta power (Fig. 2f).

Thus, in this participant, all three stimulation modalities modulated cortical activity within the theta band across distributed networks, but with differing topographies and distinct effects of stimulation parameters.

### VNS evokes distinct parameter-driver responses in low frequency spectral bands that vary among modalities and individuals

**Figure 3:**
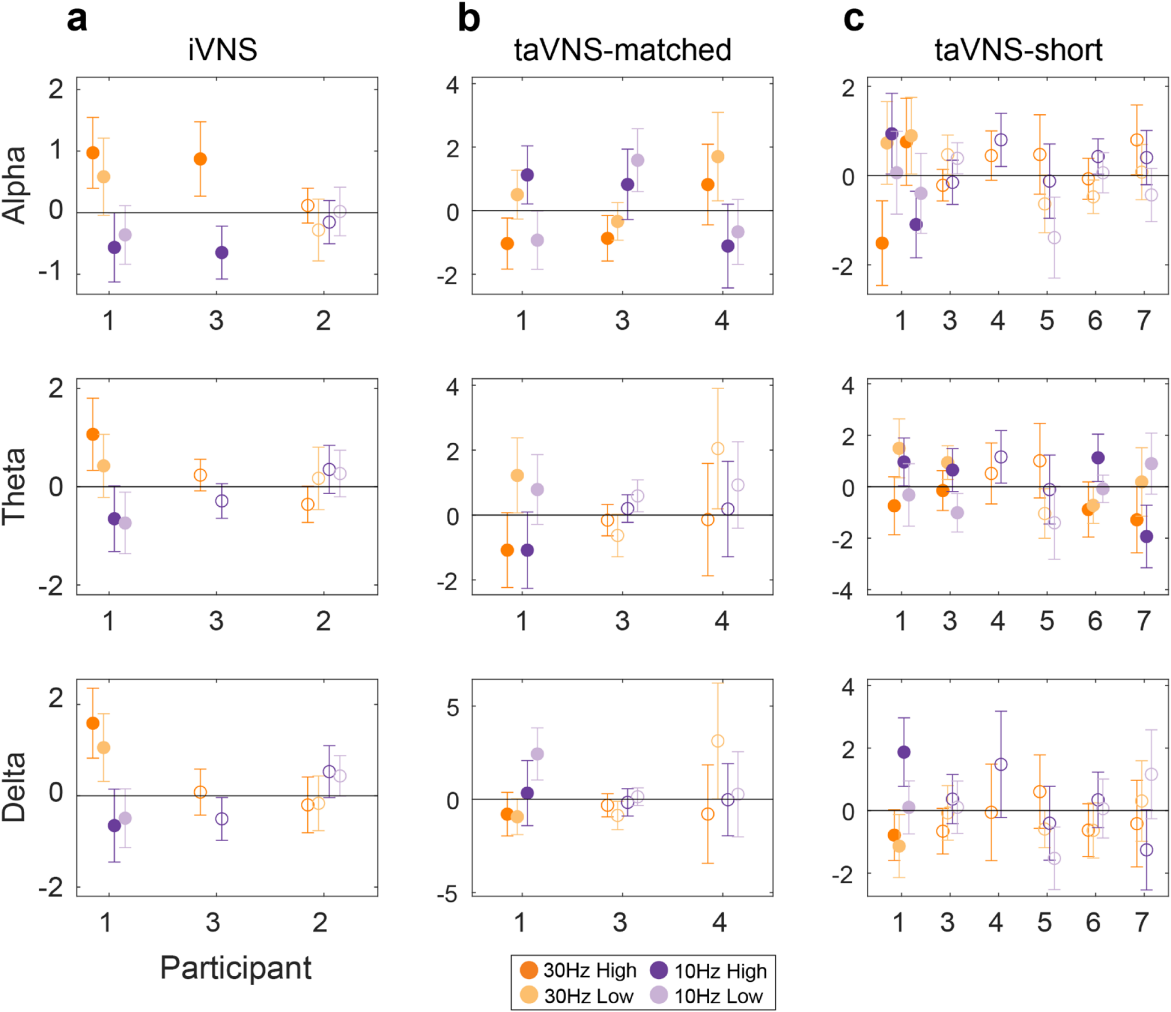
Patterns of VNS-evoked activity vary across spectral bands, stimulation modalities, and participants. (**a**) Aggregate results from cNMF and cluster-based permutation analyses for all participants that received iVNS. Each set of dots represents average spectral power in a spatiotemporal cluster. For clusters in which a significant effect was found (p < 0.05), filled dots denote mean and 95% confidence interval of power averaged over all time points identified by the cluster-based permutation analyses. Unfilled dots denote power averaged over a target window (iVNS/taVNS-matched: 2–9 s; taVNS-short: 0–2.5 s) for clusters in which no significant effect was found. For these, only the cluster with the largest difference in means between conditions is shown. Subject order is plotted for consistency across modalities. (**b**) Aggregate results for taVNS-matched. (**c**) Aggregate results for taVNS-short.

Until this point we have focused on data from one participant who received stimulation in all three modalities (iVNS, taVNS-matched, and taVNS-short), using methods that make few to no assumptions about the spatial or temporal extent of any stimulation effects, which led us to characterize the effects of stimulation at the level of broad spatiotemporal networks. We applied the same analyses on independent datasets obtained from six additional participants who performed various subsets of experiments with the VNS modalities. For each spatial/temporal cluster combination in which a significant effect was found (e.g., Fig. 2b, SC1/TC1 and SC1/TC2, p < 0.05), we computed the mean and standard error of z-scored neural activity in each condition, averaging within trials across all time points identified by the cluster-based permutation tests (Fig. 3a–c).

Using this method, six out of seven participants exhibited an effect in at least one spectral band/modality (iVNS: 2/3; taVNS-matched: 3/3; taVNS-short: 4/6). For iVNS, these effects were relatively consistent, with amplitude being greater during 30 Hz stimulation compared to 10 Hz stimulation (Fig. 3a). However, effect patterns varied across modalities, across participants within modality, and even across spectral bands within participants/modalities. For example, in taVNS-matched significant effects were found in all three participants (Fig. 3b). Yet P1 showed a main effect of amplitude in theta band and a frequency effect in delta band. While both P3 and P4 exhibited an effect of frequency in alpha, the direction of the effect was opposed between the two participants (P3: 10 Hz > 30 Hz; P4: 30 Hz > 10 Hz).

Qualitatively, the results of these unsupervised analyses indicate that the effects of stimulation modality and stimulation parameters are highly variable across participants. While bearing in mind that the limitations of our sample size as well as heterogeneity in stimulation parameters and electrode placement constrain the generalizability of any group level effects, we next attempted to identify and quantify any consistencies across modalities, spectral bands, and participants. To do this, we analyzed the data obtained from the spatiotemporal clustering using multilevel mixed effect models^35^. Mixed models enable estimation of the effects of modality and amplitude despite missing data and unequal sample sizes. Due to constraints on data acquisition, not all participants received stimulation in all three modalities and two participants did not have an amplitude contrast (P2-iVNS, due to time constraints; P4-taVNS-short, due to a low perceptual threshold). Furthermore, such models take account of the nested structure of the data (clusters within participants).

A single model was fit with fixed effects of frequency, amplitude, modality, and spectral band, and random intercepts for participant and cluster nested within participant. The dependent variable was average power within a target window for each trial, within each cluster and each participant. For iVNS and taVNS-matched, power was averaged over the peak stimulation window (2–9 s post stimulation onset). For taVNS-short, power was averaged over the time period between 0 and 2.5 s relative to stimulation onset.

We found that the effect of stimulation frequency is modality specific (Table 1). This interaction between frequency and modality appeared to be driven by a main effect of frequency in iVNS (F(1) = 31.64, p < 0.0001), with average power being greater during 30 Hz stimulation (mean = 0.21, 95% CI = [0.12 0.3]) compared to 10 Hz (mean = − 0.14, 95% CI = [− 0.24 − 0.05]). No significant frequency effects were found within the two taVNS modalities. However, in tVNS-matched, average power was greater during 10 Hz stimulation (mean = 0.16, 95% CI = [− 0.014 0.34]) compared to 30 Hz stimulation (mean = 0.01, 95% CI = [− 0.19 0.22]), while in tVNS-short average power was similar across levels of frequency (10 Hz mean + CI = 0.08 [− 0.05 0.2], 30 Hz mean + CI = 0.021 [− 0.1 0.15]).

**Table 1:** Group-level analyses. Multilevel mixed model ANOVA. Bonferroni adjusted significance level = 0.003.

Fixed effect	F-value	P-value
Frequency	1.842021	0.1747
Amplitude	1.447679	0.228
Modality	0.666705	0.5134
Band	1.067091	0.3440
Frequency:amplitude	0.020261	0.8868
Frequency:modality	**9.950729**	< 0.0001***
Amplitude:modality	3.059863	0.0469
Frequency:band	1.566740	0.2088
Amplitude:band	0.044554	0.9564
Modality:band	0.584581	0.6738
Frequency:amplitude:modality	5.048818	0.0064
Frequency:amplitude:band	0.109826	0.8960
Frequency:modality:band	0.250770	0.9093
Amplitude:modality:band	0.163854	0.9567
Frequency:amplitude:modality:band	0.639270	0.6345

### Despite distinct effects of specific parameters, responses to duty-cycle matched and short-burst stimulation exhibit some spatial and temporal similarity to iVNS responses

**Figure 4:**
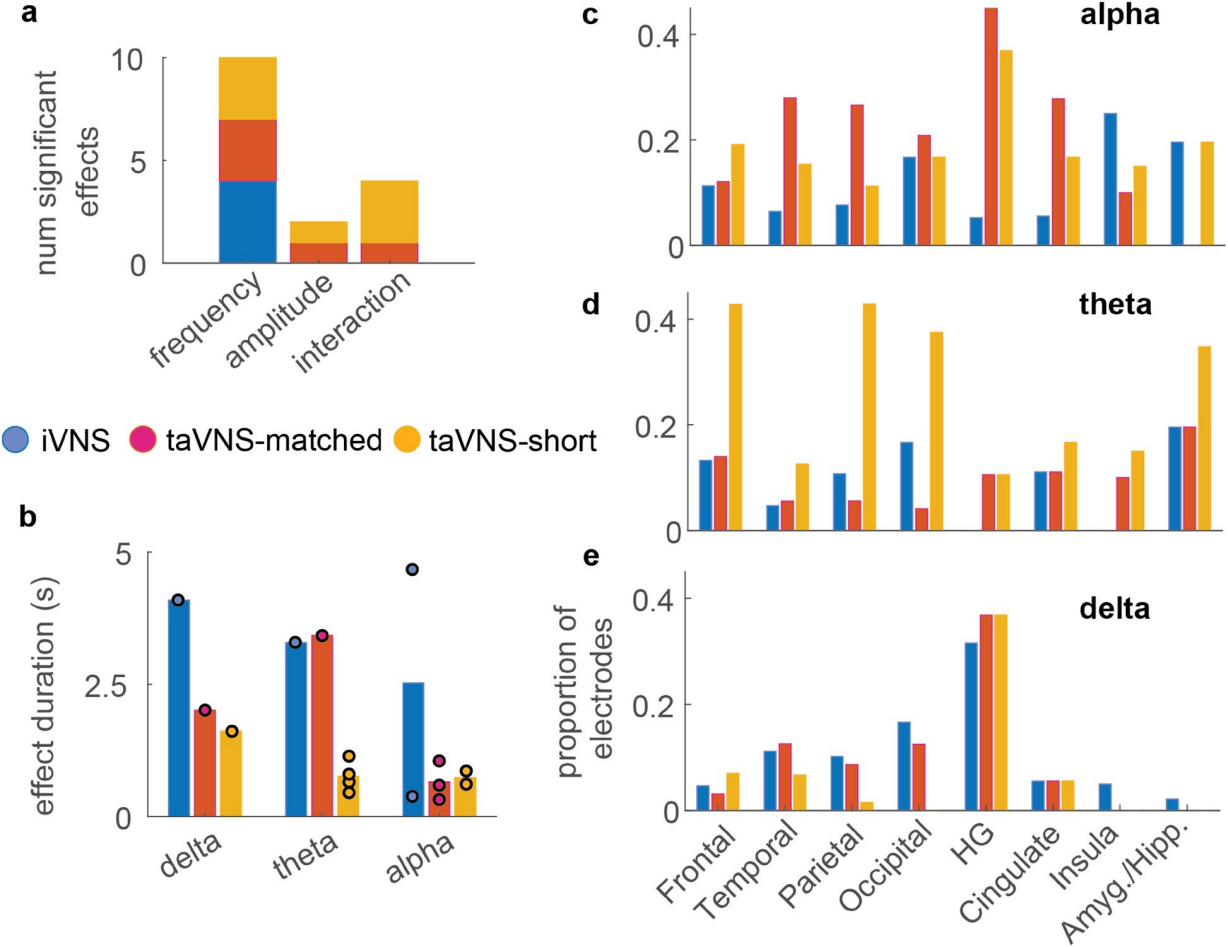
Spatial and temporal similarities between invasive and non-invasive VNS (**a**) Number of significant spatiotemporal clusters for each effect type (x-axis) and modality (color), aggregated across all spectral bands. (**b**) Total duration of significant non-parametric cluster permutation windows in each spectral band (x-axis) and modality (color). Single data points indicated by filled circles, average duration indicated by vertical bar. (**c–e**) Proportion of significant electrodes in seven regions of interest (ROIs; Amyg. = amygdala, Hipp. = hippocampus, HG = Heschl’s Gyrus) across all seven participants in the study, corresponding to spectral bands in (**c–e**) Colored bars represent, for each stimulation modality (iVNS, taVNS-matched, taVNS-short), the proportion of all electrodes within an ROI (x-axis) that were assigned to a cluster in which a significant difference between stimulation conditions was found. While proportions differ between modalities (potentially due to differences in sample size), all three are associated with changes in activity in multiple cortical and limbic areas. Cortical regions exhibit similar proportions of affected electrodes across spectral bands, whereas in subcortical structures we find fewer effects in the delta band compared to theta and alpha.

Across participants, spectral bands, stimulation modalities, and spatial clusters, we did observe some key consistencies. First, differences between stimulation-evoked responses were driven most prominently by VNS frequency (Fig. 4a). In iVNS, we found only main effects of frequency, suggesting that, for the parameter ranges used, changes in iVNS amplitude did not affect cortical activity. In contrast, frequency, amplitude, and interaction effects were observed in both taVNS modalities. Thus, within this sample, similar stimulation parameters produced consistent responses in iVNS, both across participants and spectral bands, whereas taVNS responses to similar stimulation parameters varied greatly across participants and spectral bands. However, we are unable to determine whether this is an effect of modality or instead reflects the fact that stimulation amplitude was generally higher in the iVNS modality (min = 0.75 mA, max = 2.25 mA) than in taVNS-matched (min = 0.03 mA, max = 1.6 mA) and taVNS-short (min = 0.05 mA, max = 1.9 mA).

Despite differences in parametric response patterns across modalities, we observed some similarity in the duration of effects identified by the cluster-based permutation tests (Fig. 4b). Specifically, all three modalities showed effects that were shorter than 5 s, consistent with the interpretation that the effects described here reflect acute, transient modulation of cortical activity. In addition, we found consistent patterns in the cortical regions associated with significant VNS responses within each spectral band (Fig. 4c–e). For example, in alpha, there were similar profiles of electrodes showing significant responses in frontal, temporal, Heschl’s gyrus, and cingulate cortex (Fig. 4e).

## Discussion

Using intracranial electrophysiology recordings in humans, we investigated the acute effects of parameter-specific vagus nerve stimulation on cortical activity patterns to answer three questions regarding its neurophysiological effects within and across individual participants. We found that: (1) iVNS elicited subtle changes in low frequency spectral power in local neural populations (Fig. 1). While the magnitudes of these effects were relatively small at the single electrode level, unsupervised spatiotemporal clustering revealed modulation of activity in distinct networks that spanned cortical and limbic structures. Within these networks, both invasive (iVNS) and non-invasive (taVNS) modalities evoked changes in low-frequency spectral band power (Fig. 2). (2) Within a single participant (P1), we found that the effects of stimulation on evoked power were highly dependent upon stimulation parameters of frequency and amplitude, with some combinations of parameters evoking increases in power and others decreases (Fig. 1). Furthermore, the patterns of effects, i.e., which parameters evoked increases/decreases in power, varied across modalities (Fig. 2). Across all participants, spectral bands, and modalities, we observed heterogenous patterns of effects, with some networks being affected by stimulation frequency, others by stimulation amplitude, and others by both frequency and amplitude (Fig. 3). Finally, (3) within this sample of participants, group level analyses suggested some consistency within modalities, with iVNS being modulated by frequency, taVNS-matched being affected primarily by amplitude, and taVNS-short more affected by the combination of the two (Fig. 4a). Overall, however, patterns of activity evoked by particular VNS parameters appeared to be specific to both the modality as well as the individual.

From an anatomical and neuromodulatory perspective, VNS has great potential to modulate neural activity in a wide array of cortical and subcortical networks^36–40^. VNS has been shown to induce widespread changes in activity and functional connectivity in both humans^22^ and animal models^41,42^. However, the precise extent of these network-level effects in humans had not previously been characterized with both invasive and non-invasive stimulation modalities. ECoG/SEEG provide us with sufficient spatial and temporal resolution to understand how distributed populations of neurons across widespread regions respond to VNS. Aligning with previous studies^22,41,42^, we found that variability in responses to VNS were best represented by a low-rank decomposition reflecting widespread changes in activity across cortical and limbic structures. Our findings also align with other reports of widespread changes in activity and functional connectivity in both humans^22^ and animal models^41,42^ in response to VNS. However, we also found that, within the participants who received stimulation in multiple modalities, spatiotemporal clusters were dissimilar across modalities, even between the two types of taVNS. Given the known differences between the cervical and auricular branches with regards to anatomical pathways^10,11^, histology, and nerve fiber composition^43^, it is perhaps unsurprising that different stimulation modalities produced different effects. Discrepancies may also be attributable to off-target effects during auricular stimulation^44^. It is also possible that the composition of the observed spatial clusters were not driven by stimulation modality, but instead constituted endogenous activity patterns upon which VNS acts^45–47^.

In this sample, we found that the effects of specific stimulation parameters on activity during rest were subtle and varied across individuals and experimental conditions. Even within the iVNS modality, where stimulation of the direct ascending vagus pathway was unambiguous, the effect of stimulation frequency was only consistent in two out of three participants. The small magnitude of the effects and the high degree of variability in response to comparable stimulation parameters may help to explain why results have been found to vary greatly across different VNS studies^16,19,48^. We also observed that different cortical networks exhibited different responses to VNS (Fig. 2), which may explain why prior iEEG studies that only had access to a limited number of electrodes, produced conflicting results^20,21^. However, it is important to note that this caveat may apply to our findings as well. Electrode placement was determined according to clinical care needs and varied greatly between participants (Supplementary Fig. S4). Though we were able to sample from numerous cortical structures using relatively large numbers of electrodes within patients and by pooling electrodes across participants (Fig. 4c–e), we were not able to sample exhaustively from all regions that may be affected by VNS. Furthermore, we were unable to obtain data from key subcortical structures, such as the thalamus^49^, locus coeruleus^50,51^, nucleus basalis^52^, and nucleus of the solitary tract^10,53,54^, that are pivotal to the mechanisms of VNS. Future investigations are needed to determine how the patterns of cortical effects that we observed in specific individuals and modalities may be reflected in subcortical/brainstem activity.

The high spatiotemporal resolution of the methods in the present study provide an important characterization of both within-participant patterns across VNS modalities, as well as variability across participants. Notably however, we also provide initial evidence that it is possible to achieve comparable effects across modalities using different stimulation parameters. For example, if a particular desired outcome has been shown to correlate with an increase in theta band power, this target neuromodulatory state may be attainable by application of either 30 Hz iVNS or low amplitude taVNS. Though our sample size (n = 7) is comparable to recent studies^21,22^ (though note that we sampled from a much larger set of electrodes, n = 1140), the high degree of variability we observed suggests that further work is needed to understand the multiple factors that contribute to different effects across modalities and individuals. It is also important to note that while in this study we elected to focus on acute changes in low-frequency spectral band power evoked by VNS, there are other complementary measures that were not examined here, such as functional connectivity^22^ and vagal-evoked potentials^55^, across multiple time scales (i.e., acute vs. chronic effects^4^). Indeed, by focusing on downstream cortical effects of VNS, it is difficult to use neurophysiological measures alone as a biomarker for nerve engagement.

Despite being approved and used for the treatment of epilepsy for over 20 years, iVNS is typically only 50% effective in 50% of patients^2^, and unfortunately the reasons for this variability remain unclear^56^. Given that clinical parameter settings are chosen based on a combination of empirical evidence (primarily derived from animal models^57^) and tolerability^58^, it is likely that some degree of the variance in efficacy can be attributed to differences in individual responses to specific VNS parameters^3,59^. While our study focuses only on the basic neurophysiological effects of VNS rather than on clinical outcomes, the variability in those effects coupled with the inconsistent efficacy suggests that clinical VNS parameters may need to be precisely tuned to both the particular application^25^ and the individual in order to achieve consistent therapeutic or enhancement effects. For example, extensive investigations in rat models have identified a narrow range of VNS parameters that enhance neuroplasticity^25,27,60^. Despite differences in vagal anatomy^61^, these parameters have been demonstrated to improve rehabilitation in humans^26^, though not in all cases^14^.

Even as the potential clinical, consumer, and research applications of VNS are growing rapidly, we lack a clear understanding of how stimulation works at a mechanistic level^16,62^. In a small group of participants, we constructed a detailed picture of how various brain networks are modulated by VNS, both in invasive and non-invasive paradigms. While some consistencies emerged, the overall pattern suggested that the effects of particular parameters are highly specific to the individual and the stimulation modality. As VNS continues to become more widespread as a treatment option for a range of medical conditions or as a consumer-level neuromodulation technique for various wellness and enhancement applications, investigations of individual variability in response to specific stimulation parameters will be crucial to be able to understand and predict the neurophysiological effects of VNS.

## Methods

### Standard protocol approvals, registrations, and patient consents

The Institutional Review Boards of the University of California, San Francisco, and the University of Iowa approved the study protocol. Patients provided written informed consent prior to participation and all experiments were performed in accordance with the tenets of the Declaration of Helsinki.

### Participants

Participants were seven neurosurgery patients with drug-resistant epilepsy undergoing invasive electrophysiological monitoring as part of their clinical care. Six participants were receiving care at the University of Iowa Hospitals and Clinics, and one was receiving care at the University of California, San Francisco. Depending on the clinical care objectives, participants were implanted sub-chronically with ECoG strips, grids, or SEEG depth electrodes (Table 2). Three of the seven participants had vagus nerve stimulators which had been implanted prior to the current monitoring period. In all three patients, the degree of seizure remediation provided by iVNS was deemed insufficient by the patients and the patients' neurologists. All other participants had no prior history of any type of VNS. Additional participant information is provided in Supplementary Table S2.

**Table 2:** Participant info and electrophysiological recording information.

ID	Medication (AEDs)	Seizure foci	Modalities	Data acquisition rate (samples/second)	Number of channels	Primary coverage
P1	Carbamazepine, Zonisamide	Left mesial temporal	iVNS, tVNS-matched, tVNS-short	24,000	213	Bilateral (LH Grid)
P2	Zonisamide, Lacosamide, Brivaracetam	Multiple bilateral	iVNS	24,000	120	Bilateral
P3	Lamotrigine, Lorazepam	Right mesial frontal	iVNS, tVNS-matched, tVNS-short	24,000	164	Bilateral
P4	Levetiracetam, Lacosamide, Clonazepam	Right mesial temporal	tVNS-matched, tVNS-short	24,000	239	Right
P5	none	Right amygdala	tVNS-short	4000	76	Right
P6	none	Right temporal lobe	tVNS-short	2000	238	Right
P7	Lacosamide	Left temporal	tVNS-short	3051.76	348	Left

### Electrophysiology acquisition and imaging

Intracranial electroencephalography (iEEG) recordings were obtained during quiet wakefulness. Local field potentials at each electrode were amplified and digitized according to the specifications in Table 2. All electrodes were referenced to a subgaleal electrode. Each participant underwent a preoperative magnetic resonance imaging (MRI) session to acquire structural images of the brain. After intracranial electrode implantation, participants received a computed tomography (CT) scan, which was coregistered to the MRI for individual electrode localization. The Freesurfer anatomical atlas^63^ was used to localize each electrode to an anatomical region of interest (ROI), and for visualization purposes, individual participant data were warped to a common anatomical space (cvs_avg35_inMNI152)^64^.

### Vagus nerve stimulation

Each participant received stimulation using one or more VNS modalities (iVNS, taVNS-matched, and taVNS-short; see below), depending on clinical characteristics and available testing time. We employed sub-perceptual threshold stimulation to avoid evoking somatosensory responses and to blind participants to stimulation timing and condition. Individual thresholds were determined separately for each modality.

Three patients (P1, P2, and P3) had previously been implanted with vagus nerve stimulators (AspireSR^®^ Generators Model 106, SenTiva 1000, and VNS Therapy Pulse Model 102, respectively). A neurologist trained in VNS programming oversaw all iVNS stimulation. Prior to each session, participants’ perceptual thresholds for 30 Hz stimulation were determined by slowly increasing amplitude until verbal report of being able to feel stimulation. Maximum amplitude was set at just below this threshold. We employed the shortest duty cycle available on iVNS devices: a 2 s ramp to the maximum stimulation amplitude, 7 s at maximum amplitude, and a 2 s ramp back to zero.

For taVNS, stimulation was performed using a custom-built system with a BIOPAC Constant Current Isolated Linear Stimulator. Stimulation waveforms were generated using MATLAB R2017b (MathWorks, www.mathworks.com) and transmitted to the stimulator via a National Instruments USB-6211 DAQ card. The taVNS preparation procedure is described in Fig. S1. taVNS electrodes were only affixed during taVNS-matched and taVNS-short sessions.

During taVNS thresholding, stimulation waveforms consisted of 15 biphasic square-wave pulses (250 μs pulse width) delivered at a rate of 30 Hz. Perceptual thresholds were identified using a 0.1 mA-up/0.3 mA-down staircase procedure (Supplementary Fig. S2). For all VNS modalities, the maximum amplitude for stimulation was set at just below the perceptual threshold (typically 0.2 mA below the threshold identified automatically by the staircase procedure).

Many neuromodulation studies utilize a regime of VNS in which stimulation consists of short pulse trains (< 1 s), without an amplitude ramp, and often co-occurring with a sensory stimulus or motor behavior^5^. The first taVNS-short modality datasets predated the iVNS experiments, thus the design differed slightly. In the taVNS-short modality, pulse width was set at either 100 µs or 250 µs, frequency at 10 or 25 Hz, and amplitude at levels of + 0.2, − 0.2, and − 0.4 mA relative to the participant’s perceptual threshold. These parameters were chosen based on the literature using taVNS-short paradigms^27^. As we did not expect 25 Hz to differ meaningfully from 30 Hz, the two were treated as equivalent in our analyses and visualizations. For comparability with the other modalities, our analysis included only trials with a pulse width of 250 µs and an amplitude of either − 0.2 mA (high) or − 0.4 mA (low) relative to the perceptual threshold. For three datasets (P5, P6, P7) recorded prior to the first iVNS experiment, pulse width was 150 µs (Supplementary Table S1).

During taVNS, the signal from the pulse generator was split to an analog channel on the neurophysiology recording system. Offline, taVNS pulse timing was identified using a peak-finding algorithm implemented in MATLAB R2019b (MathWorks, www.mathworks.com). This splitting procedure was not possible with iVNS, therefore we developed a novel iVNS peak finding algorithm using the signal from EKG electrodes located near the implanted pulse generator (Supplementary Fig. S3).

### Data preprocessing

For each channel, activity over an 8 ms window surrounding the center of each VNS artifact was replaced using linear interpolation^65^. Data were then filtered between 0.1 and 250 Hz using a second-order Butterworth bandpass filter. To remove line noise, notch filters at 60, 120, and 180 Hz were applied (for datasets in which sampling rate was < 20 kHz, notch filters were applied before linear interpolation as this improved artifact reduction; Table 2). Each channel was then downsampled to 512 Hz. To measure power in each spectral band, we filtered the signal within target frequency ranges using third-order Butterworth bandpass filters, applied the Hilbert transform, and downsampled the analytic amplitude timecourses to 100 Hz. For spectrograms, bandpass filters targeted 40 linearly spaced frequency bands between 1 and 20 Hz (0.5 Hz bandwidth). For canonical bands, we applied single filters with cutoff frequencies corresponding to delta (1–4 Hz), theta (4–8 Hz), and alpha (8–12 Hz). For both the LFP and each canonical band, we used visual inspection to identify bad channels, which were removed from further analysis, and ictal artifact time periods, which were set to NaN.

For iVNS and taVNS-matched, we examined activity between [− 4 11] seconds relative to stimulation onset (based on the iVNS duty cycle). The 3.5 s period between [− 4 − 0.5] seconds was designated as the baseline for each trial. Due to the high levels of noise in the data, we used single-trial full-epoch length correction^28^ to compute single-trial baseline z-score normalized power. For taVNS-short, we examined data between [− 2 2.5] seconds (1.5 s baseline = [− 2 − 0.5]).

### Unsupervised clustering

The goal of unsupervised clustering was to group electrodes with related activity and extract a prototypical time-course representing the weighted average for each cluster. Furthermore, we sought to identify spatial clusters that represented activity in specific stimulation conditions, rather than general patterns of activity across all stimulation parameters and modalities. Therefore, we first averaged the baseline-normalized trial matrix within each stimulation condition and concatenated these into an *electrode* *×* *time* matrix (Fig. 2a). We then decomposed this condition-averaged matrix into spatial clusters of activity using convex non-negative matrix factorization (cNMF^32^). This method has previously been shown to be useful for clustering neural activity based on the shape of response timecourses^33^. The algorithm computes a low rank approximation of the original data matrix *X*, according to the formula:

$$X\approx \widehat{X}=F{G}^{T},$$

where

$$F=XW.$$

The *G* matrix (*electrodes* *×* *clusters*) represents the spatial loadings of each electrode onto a particular cluster. Intuitively, *F* is the cluster centroids and *G* is the cluster membership indicators.

To determine the optimal number of clusters, we applied three common internal cluster validity algorithms, the Wemmert–Gançarski index, the Pakhira, Bandyopadhyay, and Maulik (PBM) index, and the WB-index, all of which have been shown to be effective in prototype-based clustering^66^, as well as the Xie-Beni^67^ index, a fuzzy-clustering validation technique.

After computing the cluster weights *W* using the condition-averaged matrix, we projected these weights onto the original baseline-normalized trial matrix. We first unfolded the *electrodes* *×* *time* *×* *trials* matrix into a 2D matrix by concatenating successive trials. Since this artifact-cleaned data contained NaN values, we imputed missing data points using linear interpolation. After projecting the data onto the cluster weights, the data was refolded into a *clusters* *×* *time* *×* *trials* matrix, where each cluster consisted of a weighted average over all electrodes.

### Statistical analyses

To test for condition-specific effects both at the single electrode and spatial cluster levels, we used cluster-based permutation tests^29^. For a given time series, we used ANOVA (Type III) with fixed effects of frequency, amplitude, and an interaction. Based on the significance threshold (*p* = 0.05) and the degrees of freedom, we computed a critical F-value. Contiguous samples for which the F-statistic exceeded the critical value were assigned to the same temporal cluster. For each sample within these clusters, the F-values were summed. We then performed the same procedure using 1000 random permutations, each time extracting the maximum cluster sum, to generate a null distribution. The proportion of values in the null distribution exceeding the values in the real data test set was computed to generate a *p-*value. While we qualitatively compare the size of the temporal clusters identified using this method (e.g., Fig. 4b), it is important to note that the size of the identified cluster may not represent that actual extent of the detected effect^30^.

Group level analyses were conducted using mixed effects linear regression in R with the *nlme* package^35^. Due to heterogeneity between factor levels, we fit the models to allow for different variances for each combination of participant, band, and modality. Given the factorial nature of the experimental design, we computed F-values (Type III ANOVA, deviation coded) and estimated p-values for the fixed effects with Wald-type tests (Bonferroni corrected significance = 0.003)^68^. Model results indicated the presence of a significant interaction between modality and frequency. To clarify this interaction, we fit separate “sub-models” for each modality with average power as the dependent variable. As random effects generally require > 5 levels^69^, we fit these sub-models using generalized least squares. Independent variables included frequency, amplitude, spectral band, participant ID, and where possible, the interactions between these independent variables.

## Supplementary Information

Supplementary Information.

## Data Availability

Data are available from the corresponding author upon reasonable request. All code used to generate the figures is available on GitHub at https://github.com/ChangLabUcsf/TNT_VNS_ECoG.
