# Supplementary material for: Human intracranial recordings reveal distinct cortical activity patterns during invasive and non-invasive vagus nerve stimulation
Source: Sci Rep. 2021 Nov 23;11:22780. doi: 10.1038/s41598-021-02307-x (PMC8611055; doi:10.1038/s41598-021-02307-x)
Supplement: Supplementary file 1 — Supplementary Information. [file 41598_2021_2307_MOESM1_ESM.pdf]

Human intracranial recordings reveal distinct cortical activity patterns during invasive and non-invasive vagus nerve stimulation.

William L. Schuerman, Kirill V. Nourski, Ariane E. Rhone, Matthew A. Howard, Edward F. Chang, & Matthew K. Leonard*

*Corresponding Author

SUPPLEMENTARY INFORMATION

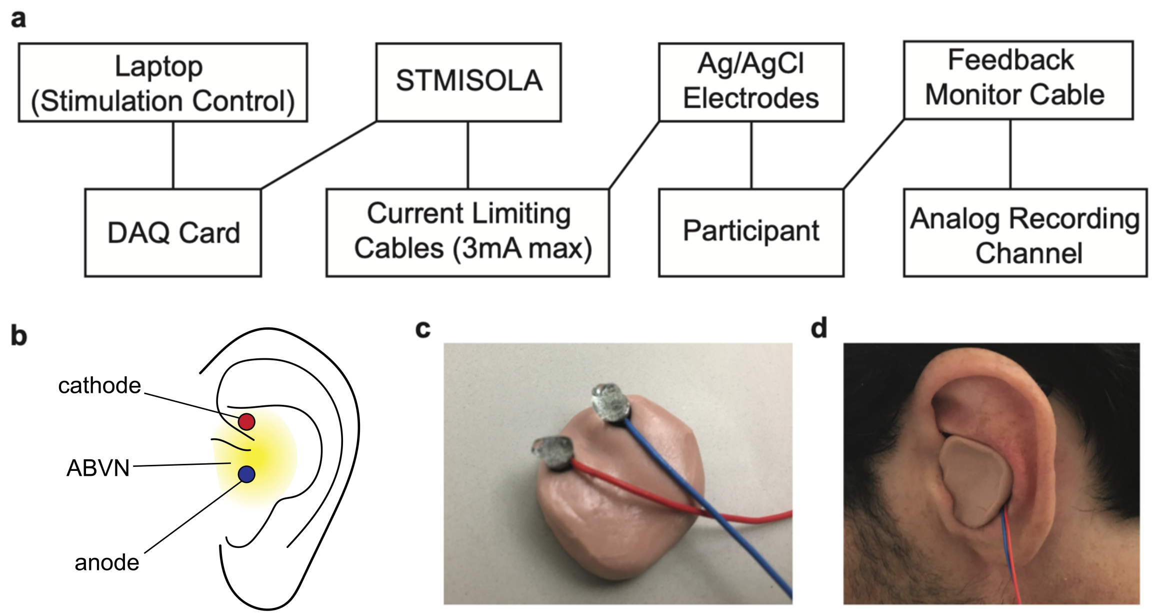

Supplementary Figure S1. Overview of tVNS system. a. Waveform generation is controlled via a task laptop running Matlab (2018b). The digital waveform signal is sent to a DAQ card (National Instruments USB-6211), with transmits the analog control signal to a BioPac STMISOLA Linear Isolated stimulator. Output from the stimulator passes through linked current limiting cables, which ensure that the biphasic stimulation waveform never exceeds a maximum of 3mA. Stimulation is delivered to the participant via two 4mm Ag/AgCl disk electrodes. A feedback monitoring cable transmits a control signal from the stimulator to an analog channel time-locked to the neurophysiological recordings. **b.** Depiction of area innervated by the auricular branch of the vagus nerve (ABVN; yellow) and target stimulation sites for cathode (red) and anode (blue) electrodes. **c**. Close-up image of stimulating electrodes. For each participant, silicone putty is used to make a mold of the ear. The electrodes are embedded in the mold at sites corresponding to the cymba concha (cathode, red wire) and cymba cavum (anode, blue wire). **d.** After cleaning and abrading the ear using an alcohol swab, the putty mold is inserted and pressed into place, ensuring that the electrodes do not move form the target sites while maintaining the comfort of the participant.

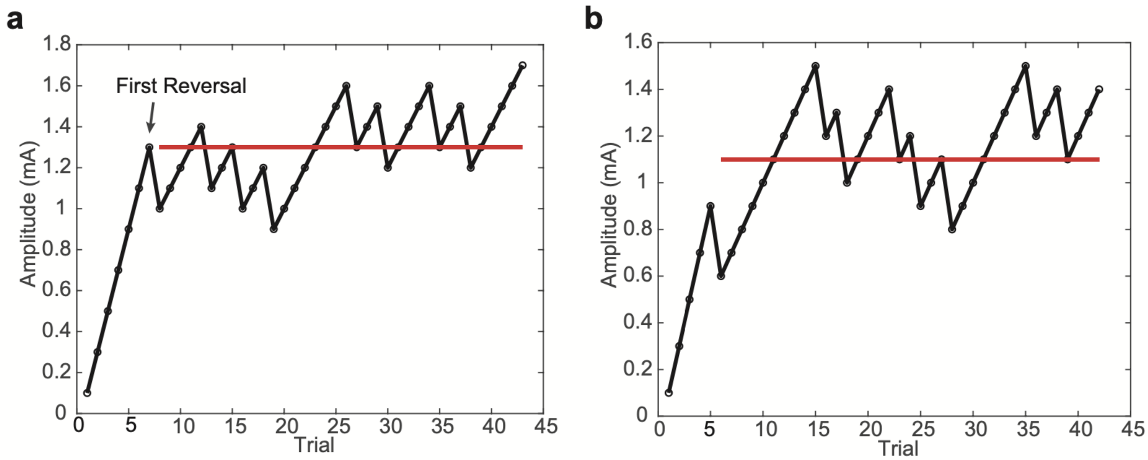

Supplementary Figure S2. Sample tVNS Perceptual Thresholds. Thresholds were obtained just prior to stimulation blocks. **a.** Perceptual threshold estimate for Participant 3. Each trial represents a pulse train of 15 square-wave biphasic pulses (30Hz, 250μs). Stimulation began at 0.1mA and was increased by 0.2mA on each trial until the participant verbally reported having felt the stimulation. At that point, amplitude was decreased by 0.3mA, and increased by 0.1mA on each subsequent trial until the participant reported feeling the stimulation, constituting a reversal. The initial estimated threshold was the average stimulation amplitude after eight reversals. As participant responses often fluctuated, the staircase results were visually inspected and if necessary the threshold was adjusted based on the judgment of the researcher. Solid red line indicates the mean amplitude (1.3mA) of all trials after the first reversal and constituted the estimate of the perceptual threshold. For this participant, the estimate was deemed reliable and stimulation was delivered at 0.2mA below this level (1.1mA; High condition) and half of that amount (0.55mA, Low condition). **b.** Same, for Participant 1. Procedure estimated the perceptual threshold at 1.1mA. However, due to the high degree of variability and the low amplitude of the first reversal compared to all others, the participant was given several 1.1mA stimulation bursts. As the participant reported not being able to feel any stimulation at this amplitude, it was utilized for the subsequent stimulation blocks.

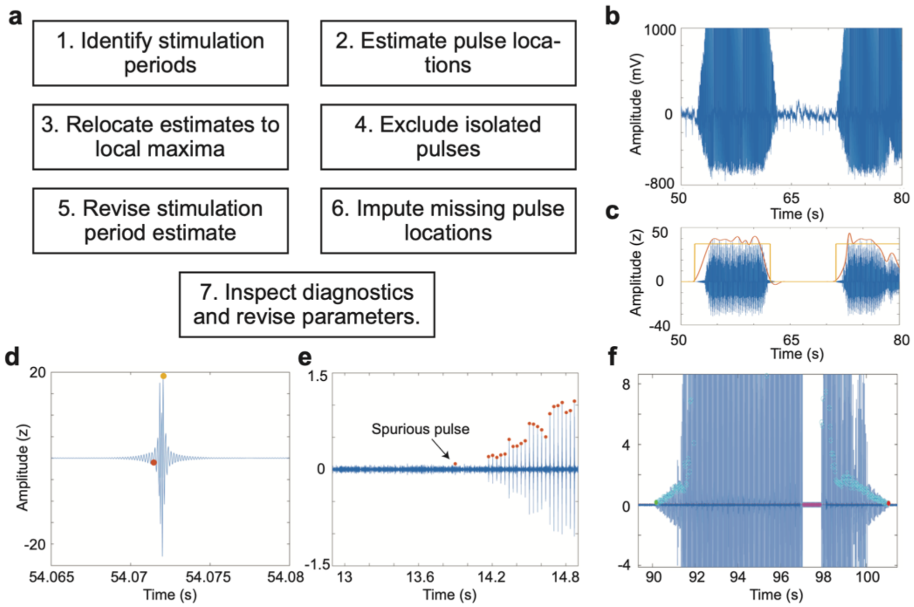

Supplementary Figure S3. Pulse artifact identification. a. Schematic of artifact identification pipeline. 1) The channel containing the clearest artifact (EKG channel for iVNS, analog channel for tVNS) is utilized for identifying pulse artifact locations. 1. The EKG channel (iVNS) is high-pass filtered (kaiser, 5000-6000Hz) to isolate the stimulation artifact from the back- ground data. For tVNS, the data is either high pass filtered at ~500Hz or z-scored, accord- ing to whichever produces the clearest signal. The envelope of the high pass filtered data is extracted, and on periods are defined as periods continuously exceeding this threshold for 6 seconds. 2. Within these periods, we slide a window corresponding to the inter-pulse-distance (IPD; based on stimulation frequency) and identify the point of maxi- mum amplitude within each window. 3. The pulse locations are revised to the maximum of a 16ms window surrounding the estimates. 4. All estimated pulse locations exceeding a distance of 1.5*IPD are discarded. 5. Based on the new pulse locations, the onsets and offsets of the stimulation periods are revised to the first and last pulse artifact in each trial. 6. In certain cases (e.g., due to amplifier saturation), data was not acquired by the recording system. In such cases, the locations of the missing artifacts are imputed based on surrounding data. 7. After performing the previous steps, diagnostics (such as the range of IPDs) are reported, and the parameters revised until estimates have been deemed accurate. **b.** Unfiltered EKG signal from participant P1 during 30Hz High stimulation. **c.** Same data after high pass filtering to reveal stimulation periods. Red line depicts envelope of signal. Yellow line depicts periods exceeding threshold delineating ON/OFF periods. **d.** Example pulse artifact. First estimate of pulse location depicted by red dot. Yellow dot indicates revised pulse location estimate based on local maximum. **e.** Example of a spurious, isolated pulse location prior to removal. **f.** Example result for a single ON period. Pulse locations marked by open cyan circles. Onset and offset marked in green and red. Magenta markers indicate pulse locations imputed through missing data.

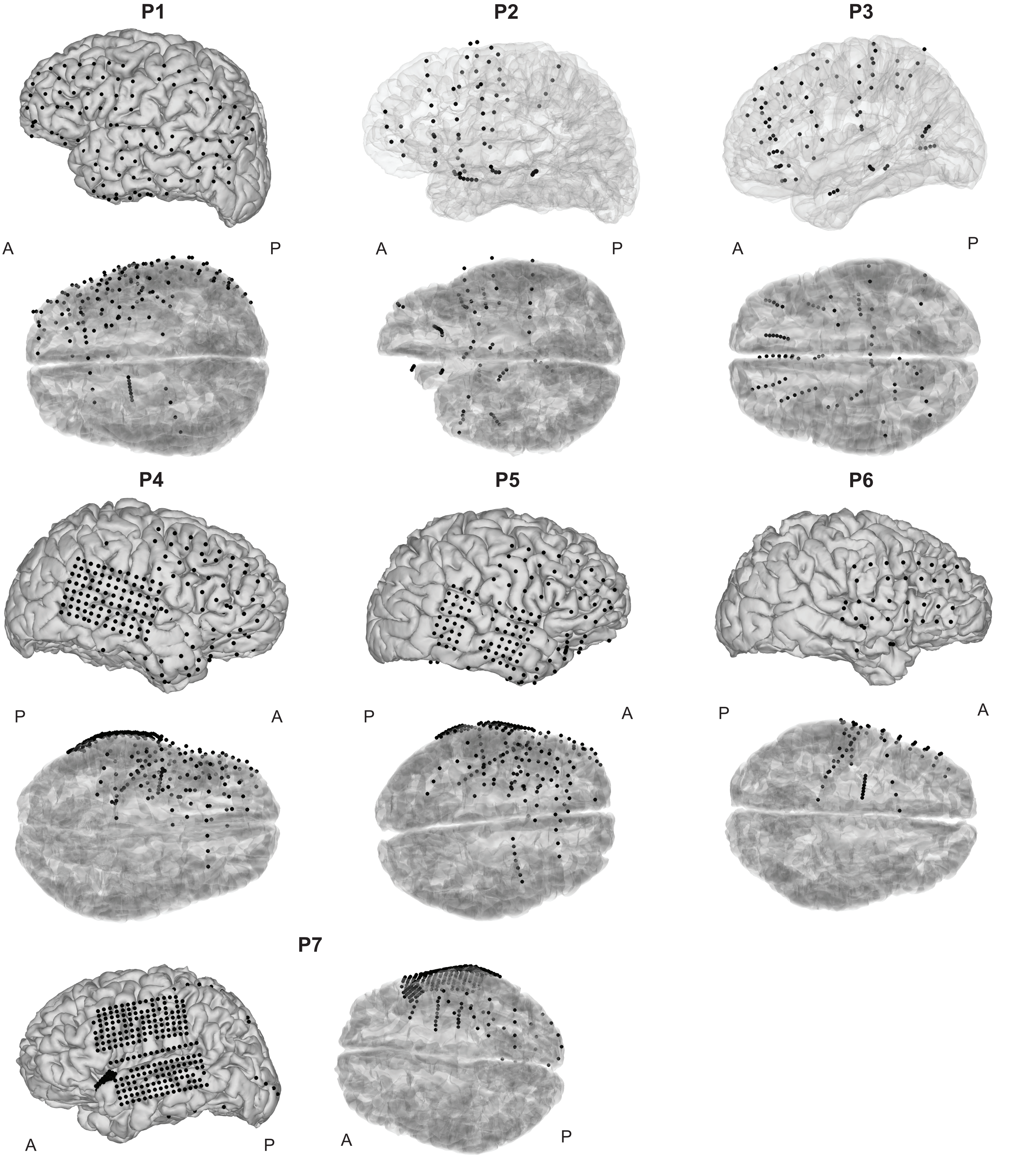

Supplementary Figure S4. Individual participant MRIs with co-registered electrode locations. For each participant, primary lateral (location of strips, grids) and ventral views are given. A and P, respectively, denote anterior and posterior locations of MRI. For SEEG participants (P2, P3), MRIs portrayed with transparency.

Supplementary Table S1. Stimulation parameters for each participant and block.

PARTI-CIPANT	BLOCK	MODALITY	PULSE WIDTH	FREQ.	AMP.	THRESHOLD ESTIMATE
**1**	1	iVNS	250μs	30	1.5	1.5
**1**	2	iVNS	250μs	30	0.75	1.5
**1**	3	iVNS	250μs	10	1.5	1.5
**1**	4	iVNS	250μs	10	0.75	1.5
**1**	5	iVNS	250μs	30	1.5	1.5
**1**	6	iVNS	250μs	30	0.75	1.5
**1**	7	iVNS	250μs	10	1.5	1.5
**1**	8	iVNS	250μs	10	0.75	1.5
**1**	9	tVNS-matched	250μs	30	1.1	1.1
**1**	10	tVNS-matched	250μs	30	0.55	1.1
**1**	11	tVNS-matched	250μs	10	1.1	1.1
**1**	12	tVNS-matched	250μs	10	0.55	1.1
**1**	13	tVNS-short	250μs	25/10	1.6/1.4	1.8
**2**	1	iVNS	250μs	30	1.5	1.5
**2**	2	iVNS	250μs	10	1.5	1.5
**2**	3	iVNS	250μs	30	1.5	1.5
**2**	4	iVNS	250μs	10	1.5	1.5
**3**	1	iVNS	250μs	30	2.25	2.25
**3**	2	iVNS	250μs	10	2.25	2.25
**3**	3	iVNS	250μs	10	1.5	1.5
**3**	4	iVNS	250μs	30	1.5	1.5
**3**	5	tVNS-matched	250μs	30	1.1	1.3
**3**	6	tVNS-matched	250μs	30	0.55	1.3
**3**	7	tVNS-matched	250μs	10	1.6	1.6
**3**	8	tVNS-matched	250μs	10	0.8	1.6
**3**	9	tVNS-short	250μs	25/10	0.4/0.2	0.6
**4**	1	tVNS-matched	250μs	30	0.06V*	1.2
**4**	2	tVNS-matched	250μs	30	0.03V*	1.2
**4**	3	tVNS-matched	250μs	10	0.2	0.2
**4**	4	tVNS-matched	250μs	10	0.1	0.2
**4**	5	tVNS-short	250μs	25/10	0.1/0.1	0.3
**5**	1	tVNS-short	150μs‡	25/10	0.7(1.9)/ 0.6(1.7)†	2.1
**6**	1	tVNS-short	150μs‡	25/10	0.1(0.3)/ 0.05(0.1)†	0.5
**7**	1	tVNS-short	150μs‡	25/10	1/0.8	1.2

Frequency denoted in Hertz, amplitude (as measured from analog channel) in milliamps. *For these two blocks, stimulation was delivered volts rather than amps. †For

these two blocks, for an unknown reason the amplitude of the analog signal did not match with logged stimulator output (given in brackets). Actual stimulation amplitude deemed to be that logged by the stimulator, not the recording system. ‡For these three patients, recorded prior to the first iVNS experiment, pulse width was set at 150μs.

Supplementary Table S2: Extended Participant Information

Participant ID	Age	Sex	iVNS	tVNS-matched	tVNS-short
1	51	M	X	X	X
2	46	M	X	o	o
3	37	M	X	X	X
4	39	F	o	X	X
5	22	F	o	o	X
6	24	M	o	o	X
7	19	M	o	o	X

Participant ID	AEDS	MRI	Seizure Focus
1	Carbamazepine (TEGretol XR) extended release tablet 200 mg, Zonisamide (ZONEGRAN) capsule 200 mg	Normal	L medial temporal
2	Zonisamide (ZONEGRAN) capsule 400 mg, Lacosamide (VIMPAT) tablet 300 mg, Brivaracetam (BRIVIACT) tablet 300 mg	Post-surgical change of R frontal lobe	Multiple frontal and temporal bilateral
3	Lamotrigine (laMICtal) tablet 200 mg, Lorazepam (ATIVAN) 2 mg/mL injection 1 mg	Encephalomalacia in R parietal and occipital lobe, gliosis in bilateral frontal lobes and in R basal ganglia	R medial posterior dorsal frontal

4	Levetiracetam (KEPPRA) tablet 1,500 mg, Lacosamide (VIMPAT) tablet 200 mg, Clonazepam (KlonoPIN) tablet 0.5 mg	Normal	R medial temporal
5	none	Normal	R temporal (uncertain medial & neocortical)
6	none	Post-surgical change of R anterior and mesial temporal lobe	R medial temporal
7	lacosamide (VIMPAT) table 400mg	Left hemispheric cortical atrophy (posterior and peri-Sylvian) in the setting of Rasmussen like encephalitis	L posterior temporal parietal region, specifically arising from posterior MTG/STG

“X” denotes that a participant received stimulation in this VNS modality,.
